# IL-33/ST2 Axis Plays a Protective Effect in *Streptococcus pyogenes* Infection through Strengthening of the Innate Immunity

**DOI:** 10.3390/ijms221910566

**Published:** 2021-09-29

**Authors:** Chih-Feng Kuo, Wei-Yu Chen, Hai-Han Yu, Yu-Hsuan Tsai, Ya-Chu Chang, Chih-Peng Chang, Nina Tsao

**Affiliations:** 1 School of Medicine, I-Shou University, Kaohsiung City 824005, Taiwan; kuo@isu.edu.tw; 2 Department of Nursing, College of Medicine, I-Shou University, Kaohsiung City 824005, Taiwan; 3 Institute for Translational Research in Biomedicine, Kaohsiung Chang Gung Memorial Hospital, Kaohsiung City 833401, Taiwan; wychen624@cgmh.org.tw; 4 Department of Biological Science and Technology, College of Medical Science and Technology, I-Shou University, Kaohsiung City 824005, Taiwan; yuhaihantaiwan@gmail.com (H.-H.Y.); vicky880818@gmail.com (Y.-H.T.); 5 Department of Medical Laboratory Science, College of Medical Science and Technology, I-Shou University, Kaohsiung City 824005, Taiwan; a0962080882@gmail.com; 6 Department of Microbiology and Immunology, College of Medicine, National Cheng Kung University, Tainan 701401, Taiwan; cp.chang711@gmail.com; 7 The Institute of Basic Medical Sciences, College of Medicine, National Cheng Kung University, Tainan 701401, Taiwan

**Keywords:** interleukin-33 (IL-33), suppression of tumorigenicity 2 (ST2), Group A *Streptococcus* (GAS), the innate immunity

## Abstract

Group A *Streptococcus* (GAS) causes invasive human diseases with the cytokine storm. Interleukin-33 (IL-33)/suppression of tumorigenicity 2 (ST2) axis is known to drive T_H_2 response, while its effect on GAS infection is unclear. We used an air pouch model to examine the effect of the IL-33/ST2 axis on GAS-induced necrotizing fasciitis. GAS infection induced IL-33 expression in wild-type (WT) C57BL/6 mice, whereas the *IL*-*33*- and *ST2*-knockout mice had higher mortality rates, more severe skin lesions and higher bacterial loads in the air pouches than those of WT mice after infection. Surveys of infiltrating cells in the air pouch of GAS-infected mice at the early stage found that the number and cell viability of infiltrating cells in both gene knockout mice were lower than those of WT mice. The predominant effector cells in GAS-infected air pouches were neutrophils. Absence of the IL-33/ST2 axis enhanced the expression of inflammatory cytokines, but not T_H_1 or T_H_2 cytokines, in the air pouch after infection. Using in vitro assays, we found that the IL-33/ST2 axis not only enhanced neutrophil migration but also strengthened the bactericidal activity of both sera and neutrophils. These results suggest that the IL-33/ST2 axis provided the protective effect on GAS infection through enhancing the innate immunity.

## 1. Introduction

Group A *Streptococcus* (GAS; *Streptococcus pyogenes*) infection causes a wide range of human diseases, extending from noninvasive pharyngitis, impetigo, and scarlet fever to invasive cellulitis, necrotizing fasciitis, and streptococcal toxic shock syndrome. GAS also induces non-purulent complications, including rheumatic fever and acute glomerulonephritis. GAS produces several virulence factors to impede the host immune system-mediated opsonophagocytosis and killing, and these virulent factors include M protein, fibronectin-binding proteins, streptococcal pyrogenic exotoxin B (SPE B), immunoglobulin-degrading enzymes, and leukocidal toxins [1]. Clinical observations have indicated that patients who suffered with GAS invasive infections, such as necrotizing fasciitis, as well as streptococcal toxic shock syndrome, produce higher inflammatory cytokines than those patients with non-invasive infections, such as scarlet fever; however, the high expression of inflammatory cytokines is also associated with a high mortality rate [2,3,4].

Interleukin-33 (IL-33) belongs to the interleukin-1 (IL-1) family of cytokines, and plays diverse roles in different infectious or inflammatory diseases [5,6,7,8]. IL-33 is expressed by different cells, including epithelial cells, endothelial cells, fibroblasts, hepatocytes, and pathogen-associated molecular pattern (PAMP) molecules, or inflammatory cytokine-activated macrophages [8,9,10,11]. IL-33 is not only a cytokine but also a nuclear factor. If the cells are quiescent, IL-33 resides within the nucleus as a transcription repressor through binding with nuclear factor kappa B (NF-κB) to block gene expression [12,13]. When the tissues are damaged, IL-33 is mostly first released by epithelial cells and endothelial cells. Therefore, it is known as an alarmin during infectious and inflammatory diseases [5,6,7,8]. Unlike other IL-1 family cytokines, which become active through caspase cleavage, the full-length IL-33, released by necrotic cells, has an active ability. However, IL-33 cleaved by caspases becomes inactive [14,15]. Recent reports have indicated that enzymes, such as proteinase 3, elastases and cathepsin G, released by neutrophils during inflammation, cleave the full-length IL-33 into the short mature form (18–21 kDa) that possesses 10–30 times the activity of the full-length one [16]. IL-33 binds to the suppression of tumorigenicity 2 (ST2), the orphan receptor of the IL-1 receptor family [5,6,7,8]. ST2 has two forms expressed by the same gene, one being the transmembrane full-length form (ST2), and the other one the soluble form (sST2) [17]. ST2 is expressed on various cell types, including natural killer (NK) cells, NKT cells, mast cells, macrophages, dendritic cells, granulocytes, type 2 innate lymphoid cells (ILC2 cells), and T_H_2 cells, but not T_H_1 cells [5,6,7,8,17,18]. Binding IL-33 to ST2 can activate NF-κB, inhibitor of NF-κB-α (IκBα), extracellular-signal-regulated kinase (ERK), p38 mitogen-activated protein kinases (p38), and Jun N-terminal kinase (JNK), and this then induces the release of inflammatory cytokines, such as interleukin-1β (IL-1β), interleukin-3 (IL-3), interleukin-6 (IL-6), tumor necrosis factor-α (TNF-α), interleukin-5 (IL-5), and interleukin-13 (IL-13) from cells [7]. The sST2, an IL-33 decoy receptor predominantly expressed by epithelial cells and fibroblasts, has a low expression in normal individuals. However, there is increased expression in patients with autoimmune diseases, myocardial infarction, and sepsis. Currently, the amount of sST2 in serum is regarded as the indicator of severity of illness [6,7,8,17].

The IL-33/ST2 axis plays diverse roles in different infectious diseases, depending on the presence of acute or chronic infection, non-invasive or invasive pathogens, and host immune tendency (T_H_1/T_H_2). Most reports indicate that the IL-33/ST2 axis drives the T_H_2 response. As the clearance of pathogens needs the T_H_1 response, the IL-33/ST2 axis may play a detrimental role in this; on the contrary, the IL-33/ST2 axis may influence a beneficial effect in controlling the strong T_H_1 response induced by pathogens [8]. IL-33 exerts protective effects in experimental sepsis-induction with cecal ligation and puncture (CLP), and while its protective mechanism is T_H_2-independent, it acts through enhancing the migration of neutrophils into the infected sites to clear bacteria [18]. GAS-induced invasive infection is associated with the cytokine storm [2,3,4]. However, the effect of the IL-33/ST2 axis on GAS infection is unclear. We used an air pouch infection model to examine GAS-induced necrotizing fasciitis [19,20]. The results indicate that *IL*-*33*- or *ST2*-knockout (KO) mice had higher mortality rates than those of wild-type (WT) mice. Surveys of the exudates from the air pouches found that there were higher bacterial loads and more dead cells in both KO mice than those of WT mice, while T_H_1 and T_H_2 cytokines were not significantly enhanced. Moreover, the predominant effector cells within the air pouches were neutrophils, while T cells, NK cells, and ILC2 cells were not involved in this model. Using the in vitro assays, we found that IL-33/ST2 affected neutrophil migration and the bactericidal activity of both the serum and neutrophils. These results suggest that IL-33/ST2 plays a protective role in the GAS-induced air pouch infective model through enhancing the innate immunity, but not by the induction of a type 2 response.

## 2. Results

### 2.1. IL-33/ST2 Axis Played a Protective Effect on GAS Infection

**Figure 1:**
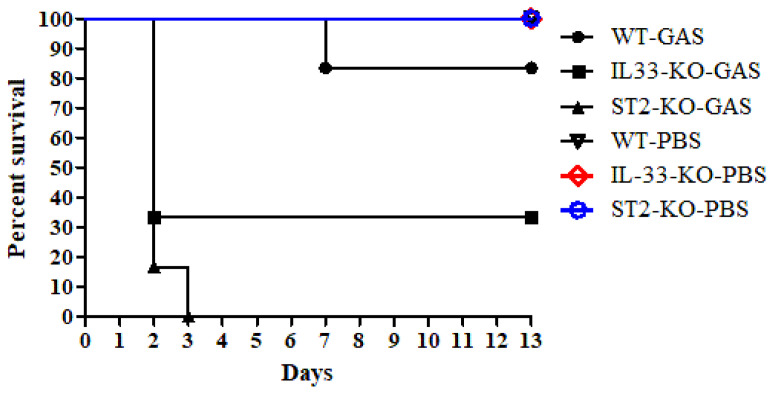
Increase in GAS-induced mortality in *IL*-*33*-KO and *ST2*-KO mice. Groups of six WT, *IL*-*33*-KO and *ST2*-KO mice were inoculated via the intra-air pouch route with 3 × 10^8^ *S. pyogenes* NZ131 cells per mouse. The animals were observed every day for a total of 13 days. Intra-air pouch injection of PBS would cause no mice death in WT, *IL*-*33*-KO and *ST2*-KO mice. Survival curves were compared for significance using the logrank test on the GAS-infected *ST2*-KO group vs. the GAS-infected B6 group (*p* = 0.0005, *p* < 0.01), and on the GAS-infected *IL*-*33*-KO group vs. the GAS-infected B6 group (*p* = 0.0417, *p* < 0.05).

**Figure 2:**
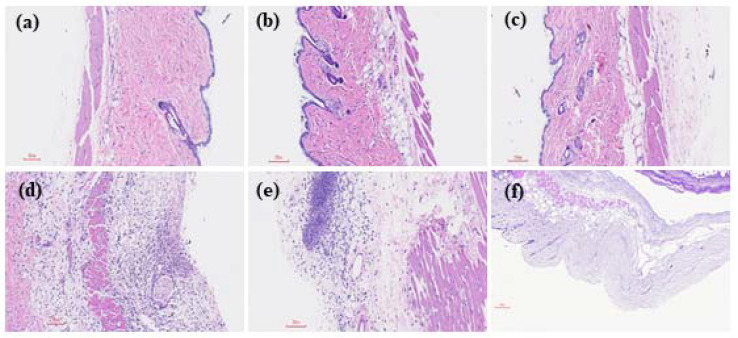
Histological examination of the skin in GAS-infected WT, *IL*-*33*-KO and *ST2*-KO mice. Groups of six WT, *IL*-*33*-KO and *ST2*-KO mice were inoculated via the intra-air pouch route with PBS or 3 × 10^8^ *S. pyogenes* NZ131 cells per mouse. The mice were sacrificed at 48 h post-infection, and then the skin sections were made and stained with H&E staining as described in Materials and Methods. The representative results are shown. (**a**): WT mouse with PBS treatment (×100); (**b**): *IL*-*33*-KO mouse with PBS treatment (×100); (**c**): *ST2*-KO mouse with PBS treatment (×100); (**d**): WT mouse with GAS treatment (×100); (**e)**: *IL*-*33*-KO mouse with GAS treatment (×100); (**f**): *ST2*-KO mouse with GAS treatment (×100).

Intra-air pouch infection of GAS caused invasive necrotizing fasciitis-like symptoms in mice [19,20]. Surveys of both exudates of air pouches and sera from GAS-infected mice found that the expression of IL-33 significantly increased after GAS infection (Supplementary Figure S1). However, the effect of the IL-33/ST2 axis on GAS infection is unclear. To address this question, we used GAS to infect *IL*-*33*- or *ST2*-knockout (KO) mice via the intra-air pouch route. As shown in Figure 1, GAS infection (3 × 10^8^ CFU/mouse) resulted in 83% (five out of six mice) survival in WT mice for 13-days observation post-infection. However, GAS infection caused 33% (two out of six mice) and 0% (0 out of six mice) survival in *IL*-*33*-KO mice and *ST2*-KO mice, respectively. In *IL*-*33*- and *ST2*-KO mice, most mice died within 2 days after infection (Figure 1). From an examination of the skin lesions, quantitated by an exudates-absorbed method, around the air pouches, we found that all *ST2*-KO mice showed skin lesions within 2 days after infection and the lesion areas were 85 ± 28 mm^2^ (*n* = 6); these lesion areas were larger than those of *IL*-*33*-KO mice (58 ± 5 mm^2^; *n* = 6, *p* < 0.01) and WT mice (29 ± 15 mm^2^; *n* = 6, *p* < 0.01) after GAS infection. The histological examination of skin lesions is shown in Figure 2. The skin lesions of GAS-infected *ST2*-KO mice appeared to show the most severe damage among the three groups, including the structure of the epidermis, dermis, and subcutaneous fat on the skin around the air pouch, which were severely damaged in GAS-infected mice (Figure 2d–f). These results indicate that the IL-33/ST2 axis provided a protective role in the GAS-infected air pouch model.

### 2.2. IL-33/ST2 Axis Affected Bacterial Burdens, Cell Infiltration and Cell Viability in Air Pouches of GAS-Infected Mice

**Figure 3:**
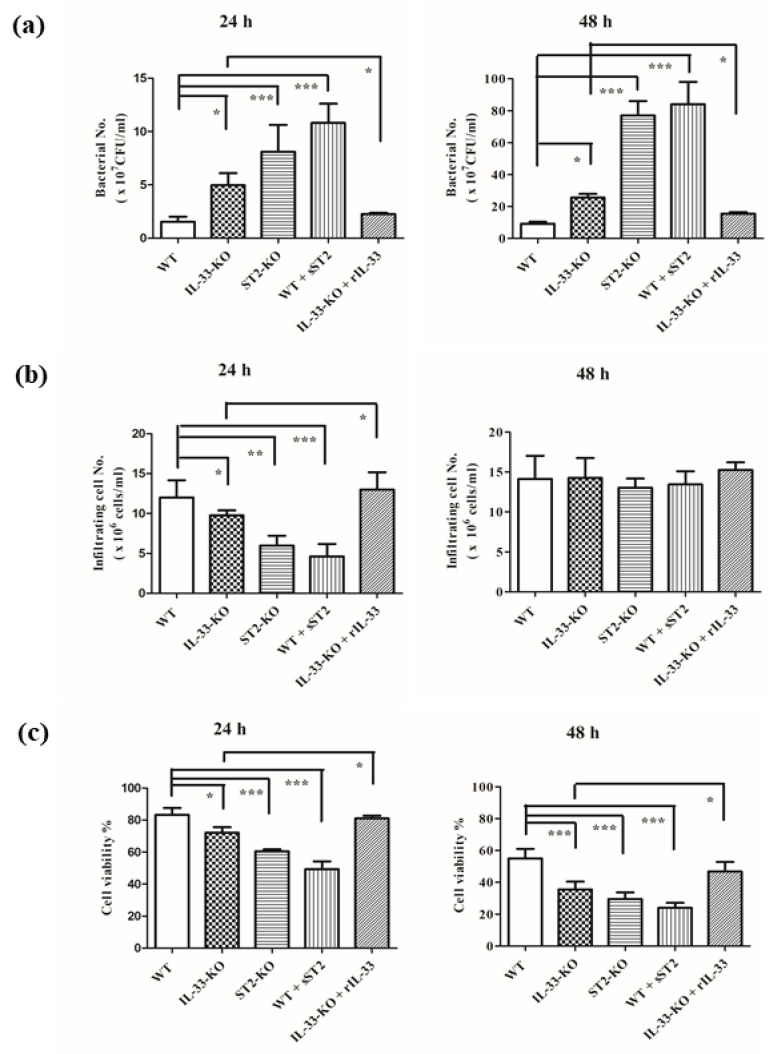
The bacterial loads, the numbers, and the cell viability of infiltrating cells in the air pouches of GAS-infected WT, *IL*-*33*-KO and *ST2*-KO mice. Groups of four WT, *IL*-*33*-KO and *ST2*-KO mice were inoculated via the intra-air pouch route with 2 × 10^8^ *S. pyogenes* NZ131 cells per mouse. In one group, *IL*-*33*-KO mice were treated with rIL-33 (1 µg per mouse) via the intra-air pouch route at 30 min before GAS infection and 24 h post-GAS infection. In the other group, WT mice were treated with sST2 fusion protein (1 µg per mouse) via the intra-air pouch route at 30 min before GAS infection and 24 h post-GAS infection. At 24 h or 48 h post-infection, exudates of the air pouch were collected, and bacterial numbers (**a**), the numbers (**b**) as well as the cell viability (**c**) of infiltrating cells were counted, as described in Materials and Methods. The results are expressed as the mean ± standard deviation. The infiltrating cells in the air pouches of WT, *IL*-*33*-KO and *ST2*-KO mice given PBS only, without GAS infection, were approximately (1.5 ± 0.3) × 10^4^ cells/mL. The infiltrating cells in the air pouch of WT mice given sST2 were approximately (2.5 ± 0.4) × 10^4^ cells/mL, while infiltrating cell numbers of B6 mice given rIL-33 were approximately (1.5 ± 0.5) × 10^6^ cells/mL. Treatment groups were compared for significance using ANOVA. * *p* < 0.05, ** *p* < 0.01, *** *p* < 0.001 compared with the GAS-infected WT group.

To examine the inflammatory conditions within the air pouches among different mice, we chose a lower infective dose (2 × 10^8^ CFU/mouse) to treat mice. At 24 h post-infection, the highest bacterial burdens were found within the air pouches of GAS-infected *ST2*-KO mice, followed by in GAS-infected *IL*-*33*-KO mice, with the lowest burden being found in the GAS-infected WT mice. The bacterial burdens in the group consisting of WT mice with an intra-air pouch with sST2/Fc fusion proteins (sST2) added 30 min before the infection, were similar to those of the GAS-infected *ST2*-KO mice. Applying recombinant mouse IL-33 (rIL-33) 30 min before infection significantly decreased the bacterial burden in GAS-infected *IL*-*33*-KO mice, when compared with GAS-infected *IL*-*33*-KO ones. Moreover, the bacterial burdens in the air pouches at 48 h post-infection were significantly increased, compared with 24 h post-infection, regardless of whether they were GAS-infected WT, *IL*-*33*-KO, or *ST2*-KO mice, and the pattern of bacterial burdens among different groups were similar to that of 24 h post-infection (Figure 3a). 

To further examine the infiltrating cell numbers within the air pouches, we found that the infiltrating cell numbers were significantly decreased in GAS-infected *IL*-*33*-KO, *ST2*-KO mice and WT mice which were given sST2 proteins at 24 h post-infection. Providing rIL-33 into the air pouches of *IL*-*33*-KO mice would restore infiltrating cell numbers at 24 h post-GAS infection (Figure 3b). This result indicates that infiltrating cells bearing ST2 receptors were the major effector cells at the early stage in the GAS-infected air pouch model. At 48 h post-infection, there were no differences in infiltrating cell numbers among different groups, possibly owing to the presence of different chemotactic molecules, other than IL-33, which were released within the air pouch at the late stage, and worked together in the recruitment of effector cells into the air pouches. 

Besides bacterial burdens and infiltrating cell numbers, we also tested the cell viability of infiltrating cells in the air pouches after GAS infection. We found that the cell viability of infiltrating cells was significantly decreased in *IL*-*33*-KO mice, *ST2*-KO mice, and WT mice given sST2 proteins post-GAS infection, when compared with that of GAS-infected WT mice at 24 h post-infection. Adding rIL-33 into the air pouches of *IL*-*33*-KO mice would restore the cell viability of infiltrating cells at 24 h post-GAS infection. As time went on, the cell viability of infiltrating cells among different groups kept falling at 48 h post-infection (Figure 3c). Based on the results mentioned above, we suggest that the absence of the IL-33/ST2 axis worsens the GAS infection through decreasing infiltrating cells, as well as their cell viability, at the early stage, and further enhancing bacterial burdens in the air pouches after GAS infection.

### 2.3. Absence of IL-33/ST2 Axis Enhanced The Expression of Interleukin-6 and Interleukin-1β but Did Not Affect the Balance of T_H_1 and T_H_2 Responses

Reports indicate that the IL-33/ST2 axis is associated with enhancing T_H_2 response in different models [8]. We examined the expression of cytokines in the air pouches after GAS infection. We tested the expression of proinflammatory cytokines, including TNF-α, interleukin-17A (IL-17A), IL-6 and IL-1β, T_H_1 cytokines such as interleukin-12 (IL-12) and interferon-γ (IFN-γ), T_H_2 cytokines such as interleukin-4 (IL-4) and IL-13, and anti-inflammatory cytokine interleukin-10 (IL-10) [21,22]. As shown in Table 1, we found that T_H_2 cytokines, both IL-4 and IL-13, in all groups were under the minimal detection limit (5 pg/mL) using the cytometric bead array (CBA) kit. T_H_1 cytokines, IL-12 and IFN-γ, and inflammatory cytokine IL-17A showed no obvious expression in the air pouch after GAS infection. The expression of proinflammatory cytokine IL-6 and IL-1β, but not TNF-α, was significantly enhanced in GAS-infected *IL*-*33*-KO mice, *ST2*-KO mice, and WT mice given sST2/Fc fusion proteins. The expression of IL-10 was slightly decreased in IL-33- or ST2-deficient mice, but there were no significant differences among different groups. These results indicate that the IL-33/ST2 axis inhibited the local inflammatory response but did not affect the balance of T_H_1 and T_H_2 in GAS-infected air pouches.

### 2.4. The Predominant Effector Cells in GAS-Infected Air Pouch Model Were Neutrophils

Previous results indicate that the absence of the IL-33/ST2 axis increased bacterial burdens, decreased both the cell viability and the numbers of infiltrating cells, and worsened the local inflammatory response in GAS-infected air pouches. We hypothesized that the infiltrating cells played an important role in controlling the local bacterial infection. The cells bearing ST2 receptors are NK cells, NKT cells, mast cells, macrophages, dendritic cells, neutrophils, eosinophils, basophils, ILC2 cells, T_H_2, and T_reg_ [6,7,8,17,18,23]. To examine the effector cells in the air pouches after GAS infection, we distinguished the populations of infiltrating cells using the staining of specific cell markers by flow cytometry. As shown in Table 2, the predominant infiltrating cells in the air pouches after GAS infection were Gr1^+^ or CD11b^+^ cells (88~96%), regardless of whether the mice were WT or KO, indicating that the major effector cells were neutrophils or monocytes [24,25]. Moreover, the microscopic observation of infiltrating cells confirmed that most effector cells were neutrophils (Supplementary Figure S2). No obvious T cells (CD3^+^) and NK cells (NK1.1^+^) were recruited into the air pouches after GAS infection. Although there were approximately 3~4% of B cells (CD19^+^ cells) in the air pouches, no statistically significant differences were found among different groups. However, the ILC2 cell (CD3^−^CD19^−^NK1.1^−^Gr1^−^CD11b^−^) [26] population was less than 1% within the GAS-infected air pouch, and there were no statistical differences among different groups. Based on the results mentioned above, we suggest that neutrophils were the predominant effector cells in GAS-infected air pouches.

### 2.5. IL-33/ST2 Axis Affected Neutrophil Migration in the In Vitro Model

**Figure 4:**
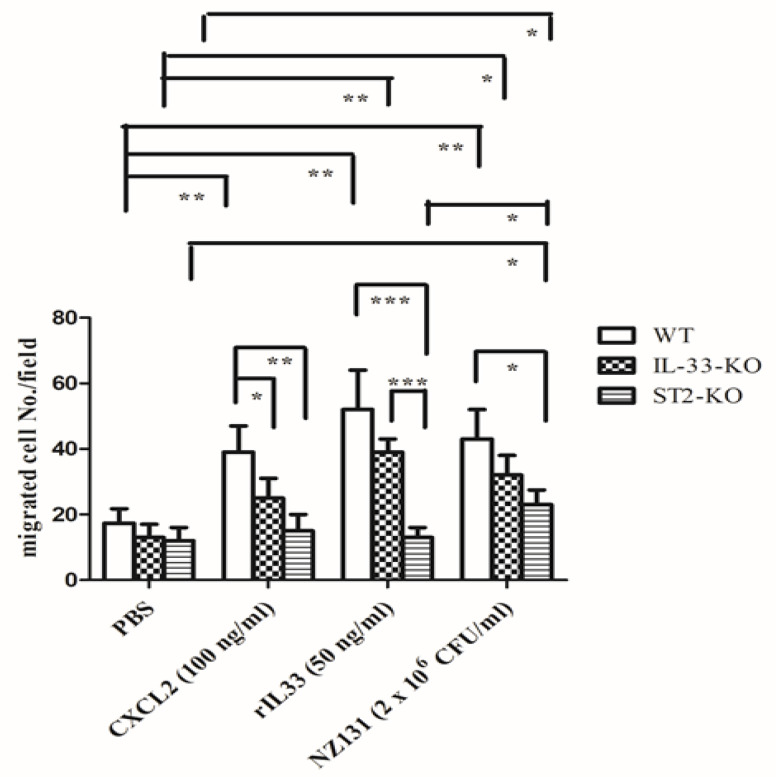
Effects of chemotaxis activities of neutrophils by different stimulants. The neutrophils of WT, *IL*-*33*-KO and *ST2*-KO mice were collected from bone marrow as described in Materials and Methods, and were stimulated by different stimulants using the transwell assay. The chemotaxis activity was examined by migrated cell numbers per HPF. Five HPFs were selected and counted in each group and the results are expressed as the mean ± standard deviation. Treatment groups were compared for significance using ANOVA. * *p* < 0.05, ** *p* < 0.01, *** *p* < 0.001 compared with the relative groups as shown in the figure.

To examine whether the IL-33/ST2 axis affected neutrophil migration, we collected neutrophils from the bone marrow of WT, *IL*-*33*-KO, and *ST2*-KO mice, and then detected them using the transwell assay. CXCL2 induced migration of neutrophils from WT mice, but not from *IL*-*33*-KO or *ST2*-KO mice, owing to the absence of the IL-33/ST2 axis which decreased CXCR2 expression on neutrophils [18]. Neutrophil migration induced by rIL-33 was more effective in WT neutrophils than in *IL*-*33*-KO neutrophils, although these findings were not statistically significant. However, rIL-33 had no recruitment effect on neutrophils from *ST2*-KO mice, indicating that the IL-33/ST2 axis clearly affected neutrophil migration (Figure 4). Mimicking in vivo infection, we used GAS as a stimulant. We found GAS itself was able to attract neutrophil migration regardless of whether the neutrophils were from WT, *IL*-*33*-KO, or *ST2*-KO mice, even though there are differences in migration efficiency among the three groups, indicating that there were other chemoattractants, distinct from the IL-33/ST2 axis, which could attract ST2-deficient neutrophil migration during infection (Figure 4).

### 2.6. Absence of IL-33/ST2 Impaired the Bactericidal Activity of Sera and Neutrophils

**Figure 5:**
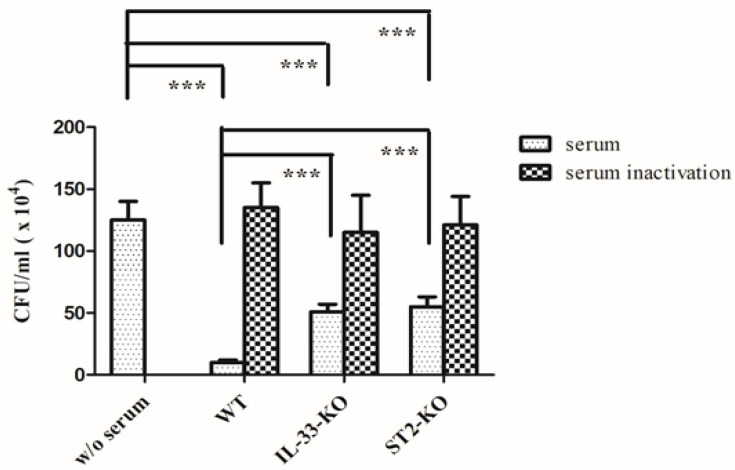
Serum killing activity against GAS. Sera collected from WT, *IL*-*33*-KO and *ST2*-KO mice were diluted 2-fold with PBS, then incubated at 4 °C or 56 °C for 30 min, and then were cultured with 1.5 × 10^4^ CFU of GAS (NZ131) for another 2 h. At the indicated time, bacteria were quantitated by plate counting. One of three experiments is represented, and the results are expressed as the mean ± standard deviation. Treatment groups were compared for significance using ANOVA. *** *p* < 0.001, compared with the relative group as shown in the figure.

**Figure 6:**
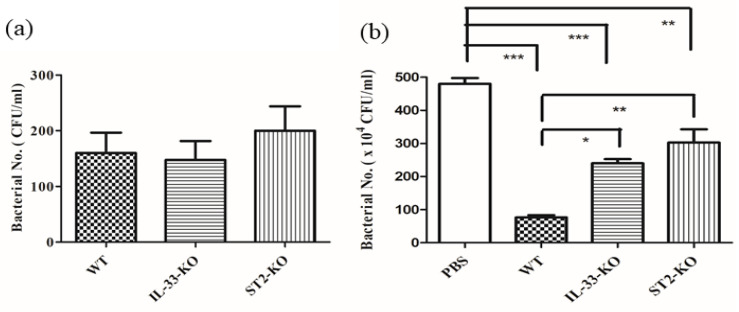
The phagocytosis activity and the bactericidal activity of neutrophils collected from WT, *IL*-*33*-KO, and *ST2*-KO mice. Bone marrow neutrophils (1.5 × 10^6^ cells) from WT, *IL*-*33*-KO, and *ST2*-KO mice were infected with GAS NZ131 cells at MOI of 0.01 for 30 min at 37 °C using a rotating mixer. After that, the extracellular bacteria were eliminated using RPMI medium containing gentamicin at 37 °C for 30 min. (**a**) After several washes by RPMI medium, the cells were lysed and intracellular viable bacteria were quantified by plating on THY agar. The bacterial numbers presented on this timepoint represent the phagocytosis of neutrophils. (**b**) Bone marrow neutrophils (1.5 × 10^6^ cells) from WT, *IL*-*33*-KO, and *ST2*-KO mice were infected with GAS NZ131 cells at MOI of 0.01 for 4 h at 37 °C using a rotating mixer. The remaining viable bacteria were counted, representing the bactericidal activity of neutrophils. Treatment groups were compared for significance using ANOVA. * *p* < 0.05, ** *p* < 0.01, *** *p* < 0.001 compared with the relative group as shown in the figure.

The bactericidal activity of sera and neutrophils were also examined. The sera, collected from different mice, were incubated with GAS for 2 h, and then the remnant bacteria were counted. The sera, pretreated at 56 °C for 30 min, lost the bactericidal activity, regardless of whether it was collected from WT, *IL*-*33*-KO, or *ST2*-KO mice. The bactericidal activity of sera, pretreated at 4 °C for 30 min, was significantly impaired in *IL*-*33*-KO and *ST2*-KO mice, compared with that of WT mice (Figure 5). To exclude the effect of the serum, the neutrophils, collected from the bone marrow, were incubated with GAS, without being sera-opsonized, and then the bactericidal activity of the neutrophils was examined. The phagocytosis activity of neutrophils from different mice was first assessed by the short-term infection of GAS. Neutrophils were infected with GAS at a multiplicity of infection (MOI) of 0.01 for 30 min, and then extracellular bacteria were killed by RPMI medium containing gentamicin. The remnant intracellular bacteria were counted and interpreted as an indicator of the phagocytosis activity of neutrophils. As shown in Figure 6a, there were no significant differences in phagocytosis among the three groups of neutrophils. In addition, neutrophils collected from WT, *IL*-*33*-KO, and *ST2*-KO mice were infected with GAS at a MOI of 0.01 for 4 h in an antibiotic-free RPMI medium, then the remnant bacteria were counted, and the bactericidal activity of the neutrophils was examined. The result indicated that the bactericidal activity of the neutrophils was significantly weakened in *IL*-*33*-KO and *ST2*-KO mice (Figure 6b). Taken together, these results suggest that the innate immunity, including that of the serum and neutrophil defense, was defective in IL-33/ST2-deficient mice.

## 3. Discussion

The incidences of invasive GAS infection are approximately 2 to 4 per 100,000 people in developed countries and 12 to 83 per 100,000 people in developing countries. The mortality rate of streptococcal toxic shock syndrome may exceed 50%, despite antibiotic administration and aggressive treatment [27]. Even though GAS vaccines based on the GAS’s virulence factor, M protein, have been developed [28,29], the safety issue due to autoreactive rheumatic heart disease by repeat immunization with recombinant M protein needs to be reassessed [30]. Hence, the therapeutic strategy of GAS invasive infection needs to be explored. Based on the clinical observations, GAS invasive infection with a high mortality is associated with the cytokine storm [2,3,4]. In this study, we examined the T_H_2 cytokine, IL-33, on a GAS-induced necrotizing fasciitis model. Our results indicate that the IL-33/ST2 axis provided a protective effect on the GAS-induced air pouch infection model, and its mechanism was through enhancing the innate immunity, including increasing the bactericidal activity of sera as well as neutrophils (Figure 5 and Figure 6b), and enhancing neutrophil migration into the infected sites (Figure 3b). Within the air pouches, we found that inflammatory cytokines, instead of T_H_1 or T_H_2 cytokines, were significantly influenced by the IL-33/ST2 axis (Table 1), indicating that the protective effect of the IL-33/ST2 axis in our model was independent of a type 2 response. This observation is similar to the experimental sepsis using the CLP model, inducing acute polymicrobial abdominal sepsis, in which IL-33 reduces mortality and improves bacterial clearance by mechanisms involved in functions of neutrophils, γδ T, and NK cells, but not T_H_2 cytokines [18,31,32,33]. However, the local environments and the virulence, as well as dosages of pathogens, seems to influence the outcomes caused by the IL-33/ST2 axis. ST2 deficiency improves the outcome of *Staphylococcus aureus*-induced septic arthritis using the intraarticular infection model [34]; in a systemic infection model induced by a lethal intravenous dose of *S. aureus*, IL-33 administration protects against lethality via ILC2-dependent type 2 cytokine production [35]; in post-influenza *S. aureus* pneumonia, IL-33 diminishes bacterial loads and mortality by an effect that does not require ILC2 cells [36]. Furthermore, IL-33 increases bacterial loads and lethality through induction of type 2 cytokines in *Legionella pneumophila*-induced pneumonia [37], while IL-33 improves local immunity during *Klebsiella pneumoniae*-induced pneumonia by a combined effect on neutrophils and inflammatory monocytes but not on B, T, NK and ILC2 cells [38].

In our air pouch infection model, we found that the local bacterial loads at the early stage played an important role in deciding the outcome. The IL-33/ST2 axis not only enhanced the number of infiltrating cells (Figure 3b), but also prolonged the survival of infiltrating cells (Figure 3c) at the early infective stage, and then accelerated the bacterial clearance (Figure 3a) and further decreased the mortality (Figure 1). Comparing *IL*-*33*-KO mice with *ST2*-KO mice after GAS infection, we found that *ST2*-KO mice had fewer infiltrating cells and lower cell viability of infiltrating cells than those of *IL*-*33*-KO mice (Figure 3b,c), indicating that ST2 receptors expressed on cells played a decisive role in influencing the survival of infiltrating cells and cell migration at the beginning of infection. Based on cell marker staining by flow cytometry (Table 2) and microscopic observation of infiltrating cells (Supplementary Figure S2), we found that neutrophils, but not T, B, NK, ILC2 cells, were the predominant effector cells within the GAS-infected air pouches. Using the transwell assay, we found that the migration of neutrophils induced by rIL-33 seemed more effective in WT neutrophils than in *IL*-*33*-KO neutrophils, although differences were not statistically significant. However, rIL-33 had no recruitment effect on neutrophils of *ST2*-KO mice, indicating that the IL-33/ST2 axis clearly affected neutrophil migration (Figure 4). However, we could not rule out that there may be other unidentified molecules, other than IL-33, that could bind to ST2 receptors to induce neutrophil migration during the early phase of infection. The directed neutrophil recruitment is orchestrated by chemoattractants, such as lipids, N-formylated peptides, complement molecules, anaphylotoxins and chemokines, produced during bacterial infection [39]. We found GAS itself was able to attract neutrophil migration using the transwell assay, regardless of whether the neutrophils were collected from WT, *IL*-*33*-KO, or *ST2*-KO mice, even though there are differences in migration efficiency among the three groups (Figure 4). This indicates that there were other chemoattractants released by GAS which could attract ST2-deficient neutrophil migration during infection. This finding explains why the numbers of infiltrating cells within the air pouches showed no differences among WT, *IL*-*33*-KO, and *ST2*-KO mice at 48 h post-infection (Figure 3b).

In addition to neutrophil recruitment, we found that bacterial clearance in the infected areas was also significantly affected by the IL-33/ST2 axis (Figure 3a). The first defense at the beginning of the bacterial infection is dependent on the complement system and phagocytes. To examine the effect of the complement system, we collected sera from different strains of mice, and the bactericidal activity in vitro was further examined. The results showed that sera from *IL*-*33*-KO and *ST2*-KO mice had weaker bactericidal activity than those of WT mice, and the remnant bacterial numbers of the KO group was five times greater than in the WT group after in vitro infection (Figure 5). Pretreatment of sera at 56 °C for 30 min destroyed the bactericidal activity of sera, regardless of whether from the WT, *IL*-*33*-KO, or *ST2*-KO group (Figure 5), indicating that the bactericidal activity of sera was complement-dependent. No literature indicates that the IL-33/ST2 axis affects the production of complement molecules. Hepatocytes can produce IL-33, and they also express ST2 receptors [40]. Whether the IL-33/ST2 axis affects complement production from hepatocytes needs further investigation. Reports have indicated that the virulence factors of GAS interfere with the bactericidal activity of complements [41,42,43,44]. As the production of complements was impaired in *IL*-*33*-KO and *ST2* KO mice, GAS may defeat the innate immunity and rapidly replicate in KO mice. Bypassing the complement effect, the bactericidal activity of neutrophils was also examined in this study. The result indicates that the bactericidal activity of neutrophils collected from KO mice was weakened, especially in those from *ST2*-KO mice (Figure 6b). A previous report indicates that the phagocytosis and production of reactive oxygen species (ROS) of phagocytes are diminished in ST2-deficient mice [45]. However, our results showed that there were no statistical differences in phagocytosis activity of neutrophils among WT and KO mice (Figure 6a). As for identifying which of the bactericidal weapons of neutrophils were affected by the IL-33/ST2 axis, this needs further examination.

In this study, we first demonstrated that the IL-33/ST2 axis was involved in protecting the mice from GAS-induced air pouch infection. Its protective mechanism was through enhancing neutrophil migration and the bactericidal activity of sera and neutrophils at the early stage of infection. These effects accelerated the bacterial clearance, following a decrease in the local inflammation, and further decreased the mortality of mice. In our air pouch infection model, no B, T, NK, or ILC2 cells were involved, and the IL-33/ST2 axis distinctly influenced the innate immunity to clear GAS infection. Based on our results shown in Figure 3, adding rIL-33 into the air pouch of *IL*-*33*-KO mice before infection significantly enhanced the bacterial clearance. The local treatment with rIL-33 provided a new therapeutic strategy on GAS invasive infection.

## 4. Materials and Methods

### 4.1. Bacterial Strain

The GAS strain NZ131 (type M49, T14) was a gift from Dr. D. R. Martin, New Zealand Communicable Disease Center, Porirua. The GAS was grown in Todd-Hewitt medium supplemented with 0.2% yeast extract (THY) (Difco Laboratories) for 12 h at 37 °C and then subcultured into fresh broth (1:50 [*vol*/*vol*]) for another 5 h to produce the log-phase bacteria. The bacterial concentration was determined with a spectrophotometer (Beckman Instruments) by measuring the optical density at 600 nm. For quantitating the exact bacterial concentration, the bacterial suspension was serially diluted with sterile phosphate-buffered saline (PBS), then 0.1 mL was poured on THY agar plates, and incubated at 37 °C overnight [46].

### 4.2. Mice

C57BL/6 (B6; WT) mice were purchased from the National Laboratory Animal Center in Taiwan. *IL*-*33*-KO and *ST2*-KO mice [47] were donated by Dr. Wei-Yu Chen, Kaohsiung Chang Gung Memorial Hospital, Taiwan, and were bred in the animal center at I-Shou University. All animals were maintained on standard laboratory chow and water ad libitum in the animal center at I-Shou University. The animals were raised and cared for in accordance with guidelines established by the Ministry of Science and Technology in Taiwan. All procedures, care and handling of the animals were reviewed and approved by the Institutional Animal Care and Use Committee at I-Shou University (ISU107026). The mice used in the experiments were 7 to 8-weeks-old (body weight 23 ± 1.0 g per mouse).

### 4.3. Air Pouch Model of GAS Infection and Evaluation of Skin Lesion

Groups of six WT, *IL*-*33*-KO, and *ST2*-KO mice were anesthetized by isoflurane inhalation and then injected subcutaneously with 1 mL of air to form an air pouch one day before the infection. One day later, 0.1 mL of bacterial suspension containing 3 × 10^8^ *S. pyogenes* NZ131 cells was inoculated into the air pouch [48]. The animals were observed every day for a total of 13 days. Survival curves were then determined. At 48 h post-infection, the degrees of skin lesion were quantitated by an exudates-absorbed method, which is based on the premise that the more severe the skin damage, the more that the exudates will effuse. A Kimwipes (Kimberly-Clark Global Sales, Inc., Roswell, GA, USA) paper was attached to the air pouch area, and the wet area of the paper was then gauged [49]. The average lesion area in each group was generated by examination of skin lesions from six mice. After that, the skin lesion tissue around the air pouch was excised, fixed in 3.7% formaldehyde, and embedded in paraffin. The 5-µm-thick tissues were sliced and stained with hematoxylin and eosin. In some experiments, 0.1 mL of mouse rIL-33 (R&D Systems) (1 µg per mouse) and sST2 protein (R&D Systems) (1 µg per mouse) were injected into the air pouches of *IL*-*33*-KO mice and WT mice, respectively, at 30 min before GAS infection or 24 h post-infection.

### 4.4. Examination of the Inflammation and the Populations of Infiltrating Cells within the Air Pouches

Groups of four WT, *IL*-*33*-KO, and *ST2*-KO mice were inoculated with 0.1 mL of bacterial suspension containing 3 × 10^8^ *S. pyogenes* NZ131 cells into the air pouch. At 48 h post-infection, the mice were anesthetized by isoflurane inhalation, then the blood was collected by the cardiac puncture, and the exudates of the air pouch were collected by injecting 1 mL of sterile PBS into the air pouch and aspirating the exudates by syringe with an 18-gauge needle. The blood and exudates were examined by a capture IL-33 enzyme-linked immunoassay (ELISA) kit (R&D Systems). For evaluation of the inflammation after GAS infection in the groups of four WT, *IL*-*33*-KO, and *ST2*-KO mice, WT mice given sST2 proteins, and *IL*-*33*-KO mice given rIL-33, were inoculated with 0.1 mL of bacterial suspension containing 2 × 10^8^ *S. pyogenes* NZ131 cells into the air pouch. At different times after infection, the exudates of the air pouch were collected. For quantitation of bacterial numbers within the air pouch, 10 µL of the exudates were lifted, underwent serial dilution, poured (0.1 mL) in THY agar plates, and incubated at 37 °C overnight. The number of CFU of *S. pyogenes* was then quantitated and expressed as the mean ± standard deviation. The remnant exudates were then centrifuged for 10 min at 1200 rpm. The sediment containing the infiltrating cells was collected, and the number and viability of the infiltrating cells were further determined by an eosin Y exclusion assay [49]. The number and the cell viability of infiltrating cells were then quantitated and expressed as the mean ± standard deviation. In addition, infiltrating cells were further stained by antibodies of different cell markers, including CD3ε monoclonal antibody (145-2C11) conjugated with APC (eBioscience 17-0031-83), CD19 monoclonal antibody (eBio1D3) conjugated with PE (eBioscience 12-0193-82), NK1.1 monoclonal antibody (PK136) conjugated with PE (eBioscience 12-5941-81), Gr1 (Ly-6G/Ly-6C) monoclonal antibody (RB6-8C5) conjugated with FITC (eBioscience 11-5931-85), CD11b monoclonal antibody (M1/70) conjugated with PerCP-Cy™5.5 (BD Pharmingen 550993). The percentages of the cell marker staining were expressed as the mean ± standard deviation. Moreover, the infiltrating cells were further assessed by the cytospin and Liu stain, and were then observed by microscope. The expression of cytokines, containing TNF-α, IL-17A, IL-6, IL-1β, IL-12, IFN-γ, IL-4, IL-13 and IL-10, in the supernatants of exudates was also examined using a cytometric bead array kit (BD Biosciences). In this detection kit, the limit of cytokine detection was 5 pg/mL. The concentrations of cytokines in the air pouches of mice were quantitated and expressed as the mean ± standard deviation.

### 4.5. Chemotaxis Assay of Mouse Neutrophils Isolated from Bone Marrow

WT, *IL*-*33*-KO, and *ST2*-KO mice were sacrificed, and femur and tibia from both hind legs were removed and cleared of remaining soft tissue attachments. After that, the extreme distal tip of each extremity was cut off and the bones flushed with HBSS medium (with calcium, magnesium, Gibco) containing 0.5% bovine serum albumin and 1% glucose (SigmaAldrich) and 20 mM HEPES (Merck-SigmaAldrich) at a pH of 7.2, using a 25G needle. After, the bone marrow was flushed on ice, then centrifuged at 1300 rpm for 10 min, and the sediment containing the cells was treated with the RBC lysing buffer (Sigma) to eliminate RBCs. The remaining cells were suspended in 2 mL of HBSS medium and treated on a three-layer Percoll (GE Heathcare) density gradient, 72%, 63%, and 50%, respectively, diluted in HBSS, and centrifuged for 25 min at 3000 rpm at 4 °C. Neutrophils were harvested from the 63%/72% interface after carefully removing the cells from the upper phases [50]. The cell preparations contained >90% neutrophils as determined by visual counting. The neutrophil populations with viability over 95%, as assessed by trypan blue exclusion, were used to conduct the chemotaxis assay. The hanging cell insert with 8-μm membrane (Millipore) was hung in a 24-well culture plate (Nunc) to examine the chemotaxis of neutrophils [18]. Neutrophils (1 × 10^6^ cells/mL) were added in the hanging cell insert, and different stimulants, such as PBS, CXCL2 (100 ng/mL), mouse rIL-33 (50 ng/mL), or GAS NZ131 cells (2 × 10^6^ CFU/mL), were added to the 24-well culture plate. After a 1 h culture, the hanging cell insert was removed, and the membrane was cut, fixed, and stained with 0.05% crystal violet (Sigma). The bottom layer of the membrane was examined by microscope, and the number of cells on each high-power field (HPF) was counted, and then cells of 5 HPFs from each group were quantitated and expressed as the mean ± standard deviation.

### 4.6. Serum Bactericidal Assay

The sera from WT, *IL*-*33*-KO, and *ST2*-KO mice were collected. The sera, diluted 2-fold with PBS, were incubated at 56 °C for 30 min to inactivate the complement system. GAS NZ131 cells (1.5 × 10^4^ CFU in 100 µL PBS) were incubated with 5 µL of diluted sera, pretreated for 30 min at either 4 °C or 56 °C, and at 37 °C for 2 h using a rotating mixer. After that, the remaining viable bacteria were diluted with PBS, and then quantified by plating on THY agar plates. One of three experiments was represented, and the results were expressed as the mean ± standard deviation.

### 4.7. Phagocytosis and Bactericidal Assay of Neutrophils

Neutrophils from the bone marrow were separated using a Percoll (GE Heathcare) density gradient, as described previously. Neutrophils (1.5 × 10^6^ cells in 0.2 mL RPMI medium, Gibco) from WT, *IL*-*33*-KO, and *ST2*-KO mice were infected with GAS (50 µL) at a MOI of 0.01 for 30 min at 37 °C using a rotating mixer. After that, cell suspensions were centrifuged at 6000 rpm for 10 min at 4 °C. The supernatants were discarded, and the sediment cells were cultured with RPMI medium containing gentamicin (50 µg/mL) at 37 °C for 30 min to kill extracellular bacteria. After several washes by RPMI medium, the cells were lysed, and intracellular viable bacteria were then quantified by plating on THY agar plates as described previously [48]. The bacterial numbers presented on this timepoint represent the phagocytosis of neutrophils. 

Neutrophils (1.5 × 10^6^ cells in 0.2 mL RPMI medium, Gibco) from WT, *IL*-*33*-KO, and *ST2*-KO mice were infected with GAS (50 µL) at a MOI of 0.01 for 4 h at 37 °C. After that, cell suspensions were centrifuged, the supernatants were collected, the sediment cells were lysed. The intracellular viable bacteria and remaining viable bacteria in the supernatants were further quantified by plating on THY agar plates as described previously [48]. The bacterial numbers presented on this timepoint represent the bactericidal activity of neutrophils.

### 4.8. Statistics

The statistical analysis was conducted using Prism 5.0 software (GraphPad Software, San Diego, CA, USA). In Figure S1, treatment groups were compared for significance using the *t*-test. For the mouse survival in Figure 1, survival curves were compared for significance using the logrank test. The data shown in Figure 3, Figure 4, Figure 5 and Figure 6 and the tables were compared for significance using ANOVA. Statistical significance was set at * *p* < 0.05, ** *p* < 0.01, ***p < 0.001.

## Figures and Tables

**Table 1:** Cytokine expression in exudates of mouse air pouches after GAS infection.

Treatment	TNFα	IL-17A	IL-6	IL-1β	IL-12	IFN-γ	IL-10 (pg/mL)
WT-PBS	<5 ^a^	<5 ^a^	<5 ^a^	<5 ^a^	<5 ^a^	<5 ^a^	<5 ^a^
WT + rIL-33	6 ± 1	<5 ^a^	<5 ^a^	<5 ^a^	<5 ^a^	<5 ^a^	7 ± 2
WT + sST2	6 ± 1	<5 ^a^	<5 ^a^	<5 ^a^	<5 ^a^	<5 ^a^	<5 ^a^
WT-GAS	891 ± 86	<5 ^a^	4601 ± 875	724 ± 98	7 ± 2	7 ± 2	66 ± 19
*IL*-*33*-KO-GAS	498 ± 95 *	<5 ^a^	6150 ± 345 *	1324 ± 297 *	9 ± 4	<5 ^a^	53 ± 8
*ST2*-KO-GAS	501 ± 101	6 ± 1	7325 ± 215 *	1678 ± 112 *	8 ± 3	<5 ^a^	28 ± 4
WT-GAS + sST2	489 ± 198	7 ± 2	7370 ± 550 *	1860 ± 276 *	9 ± 1	<5 ^a^	21 ± 6
*IL*-*33*-KO-GAS + rIL-33	798 ± 120	<5 ^a^	4150 ± 621	813 ± 113	8 ± 2	6 ± 2	68 ± 13

Groups of four B6, *IL*-*33*-KO mice and *ST2*-KO mice were inoculated via the intra-air pouch route with 2 × 10^8^ *S. pyogenes* NZ131 cells per mouse. In one group, *IL*-*33*-KO mice were given rIL-33 (1 µg per mouse) via the intra-air pouch route at 30 min before GAS infection. In the other group, B6 mice were treated with recombinant sST2/Fc fusion protein (1 µg per mouse) via the intra-air pouch route at 30 min before GAS infection. In control groups, B6 mice were given rIL-33 (1 µg per mouse) or sST2/Fc fusion protein (1 µg per mouse) only via the intra-air pouch route. At 24 h post-infection, exudates of the air pouch were collected, and cytokine expressions were examined using a cytometric bead array (CBA) kit. The results were expressed as the mean ± standard deviation. The statistical analysis was performed using repeated-measures ANOVA. * *p* < 0.05 compared with results for GAS-infected WT mice. ^a^ The minimal detection concentration of the cytokine using a CBA kit.

**Table 2:** Analysis of cell populations of infiltrating cells in the air pouches of mice after GAS infection.

Treatment	CD3	CD19	NK1.1	Gr1	CD11b (%)
WT-GAS	0.38 ± 0.01	3.93 ± 0.3	0.24 ± 0.03	90 ± 5.2	95 ± 1.0
*IL*-*33*-KO-GAS	0.42 ± 0.03	3.71 ± 2.4	0.70 ± 0.10	89 ± 5.1	93 ± 2.8
*ST2*-KO-GAS	0.20 ± 0.03	3.10 ± 0.3	0.15 ± 0.02	90 ± 3.5	93 ± 4.2
WT-GAS + sST2	0.10 ± 0.05	2.94 ± 0.7	0.10 ± 0.10	88 ± 5.2	96 ± 2.1
*IL*-*33*-KO-GAS + rIL-33	0.28 ± 0.05	3.53 ± 0.4	0.33 ± 0.10	93 ± 3.4	96 ± 2.6

Groups of four B6, *IL*-*33*-KO mice and *ST2*-KO mice were inoculated via the intra-air pouch route with 2 × 10^8^ *S. pyogenes* NZ131 cells per mouse. In one group, *IL*-*33*-KO mice were given rIL-33 (1 µg per mouse) via the intra-air pouch route at 30 min before GAS infection. In the other group, B6 mice were treated with recombinant sST2/Fc fusion protein (1 µg per mouse) via the intra-air pouch route at 30 min before GAS infection. In control groups, B6 mice were given rIL-33 (1 µg per mouse) or sST2/Fc fusion protein (1 µg per mouse) only via the intra-air pouch route. At 24 h post-infection, exudates of the air pouch were collected, and the cell populations of infiltrating cells were examined using the specific cell marker staining. The results were expressed as the mean ± standard deviation. The infiltrating cells in control groups were counted and analyzed by the specific cell marker staining. The predominant infiltrating cell populations in both control groups were also Gr1^+^ or CD11b^+^ cells.

## Data Availability

The datasets generated during and/or analyzed during the current study are available from the corresponding author on reasonable request.

## Supplementary Materials

The following are available online at https://www.mdpi.com/article/10.3390/ijms221910566/s1, Figure S1: The IL-33 expression in GAS-infected B6 mice, Figure S2: The morphology of the infiltrating cells in the air pouches.
