# Supplementary material for: IL-33/ST2 Axis Plays a Protective Effect in Streptococcus pyogenes Infection through Strengthening of the Innate Immunity
Source: Int J Mol Sci. 2021 Sep 29;22(19):10566. doi: 10.3390/ijms221910566 (PMC8509005; doi:10.3390/ijms221910566)
Supplement: Supplementary file 1 [file ijms-22-10566-s001.zip › ijms-1398785-S1.pdf]

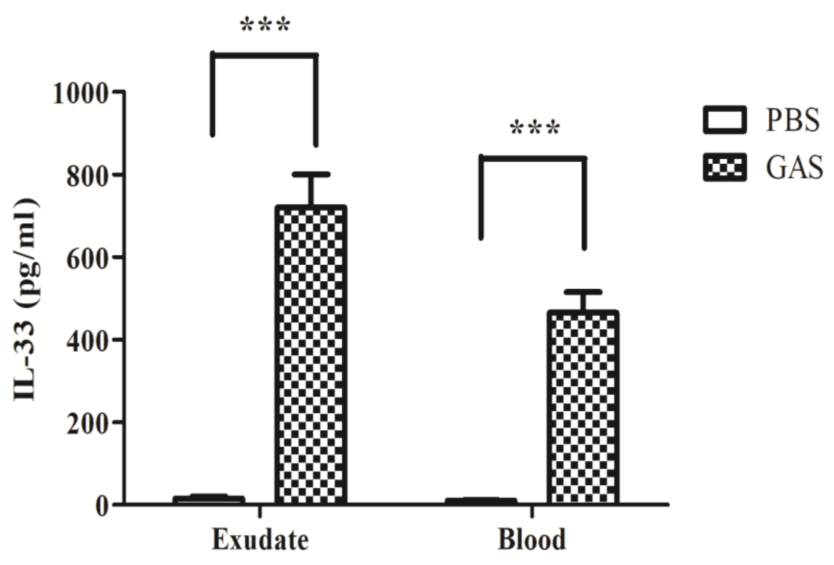

Figure S1. The IL-33 expression in GAS-infected B6 mice. Groups of three B6 mice were inoculated via the intra-air pouch route with PBS or 3×10^8 *S. pyogenes* NZ131 cells per mouse, as described in Materials and Methods. At 48 h post-infection, mouse exudates and blood were collected, and the concentrations of IL-33 were determined by a capture IL-33 ELISA kit. The concentration of IL-33 was expressed as the mean ± standard deviation. *** $P < 0.001$ compared with the PBS group.

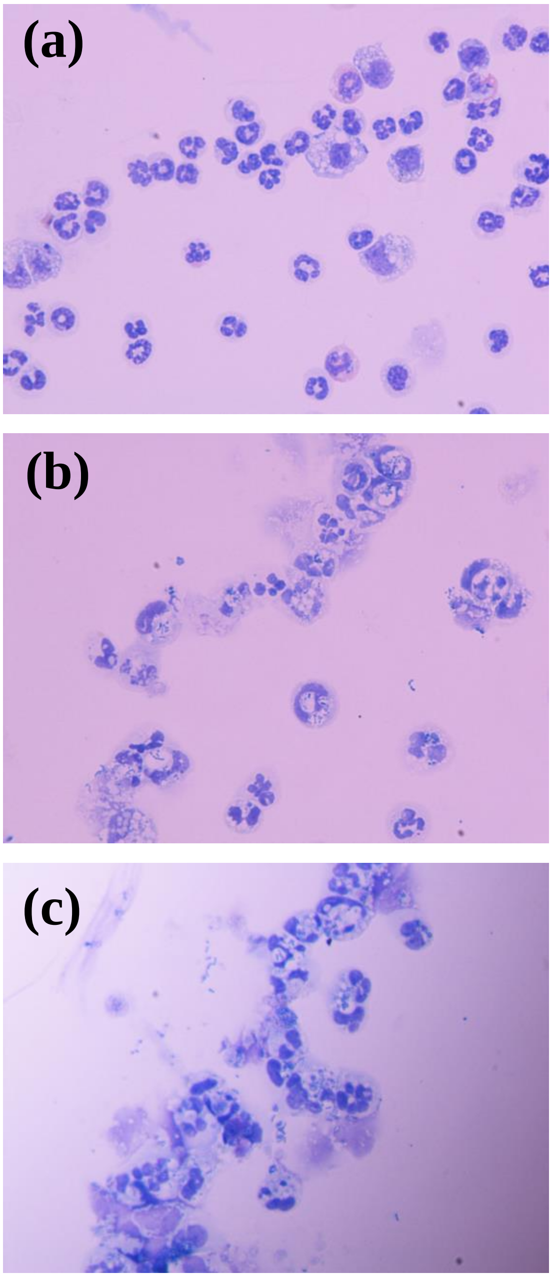

Figure S2. The morphology of the infiltrating cells in the air pouches. Groups of four WT, *IL-33*-KO or *ST2*-KO mice were inoculated via the intra-air pouch route with 2×10^8 *S. pyogenes* NZ131 cells per mouse. At 24 h post-infection, exudates of the air pouch were collected, and the infiltrating cells were assessed by the cytospin and Liu stain, and then were observed by microscope. The representative results are shown. a: WT mouse with GAS treatment (× 400); b: *IL-33*-KO mouse with GAS treatment (× 400); c: *ST2*-KO mouse with GAS treatment (× 400).
